# Platelet secretion: From haemostasis to wound healing and beyond

**DOI:** 10.1016/j.blre.2014.10.003

**Published:** 2015-05

**Authors:** Ewelina M. Golebiewska, Alastair W. Poole

**Affiliations:** Medical Sciences Building, School of Physiology and Pharmacology, University of Bristol, University Walk, BS8 1TD Bristol, UK

**Keywords:** Platelets, Secretion, SNARE proteins, Thrombosis, Haemostasis, Cancer metastasis, Inflammation

## Abstract

Upon activation, platelets secrete more than 300 active substances from their intracellular granules. Platelet dense granule components, such as ADP and polyphosphates, contribute to haemostasis and coagulation, but also play a role in cancer metastasis. α-Granules contain multiple cytokines, mitogens, pro- and anti-inflammatory factors and other bioactive molecules that are essential regulators in the complex microenvironment of the growing thrombus but also contribute to a number of disease processes. Our understanding of the molecular mechanisms of secretion and the genetic regulation of granule biogenesis still remains incomplete. In this review we summarise our current understanding of the roles of platelet secretion in health and disease, and discuss some of the hypotheses that may explain how platelets may control the release of its many secreted components in a context-specific manner, to allow platelets to play multiple roles in health and disease.

## 1 Introduction

Platelets have been known to contribute to thrombosis and haemostasis since their first identification by Bizzozero in the 1880s [1]. Recent evidence suggests that their functions extend beyond the immediate environment of the thrombus and platelets have been implicated in a number of other physiological responses aimed at safeguarding the integrity of the vessel. On the other hand, their properties as haemostatic and inflammatory cells can result in disease states under certain conditions. Platelets can ‘communicate’ with each other and with other cells via a range of bioactive substances secreted from their intracellular granules. In this review, the contribution of platelet secretion to those processes will be discussed.

## 2 Platelet activation cascade

**Fig. 1:**
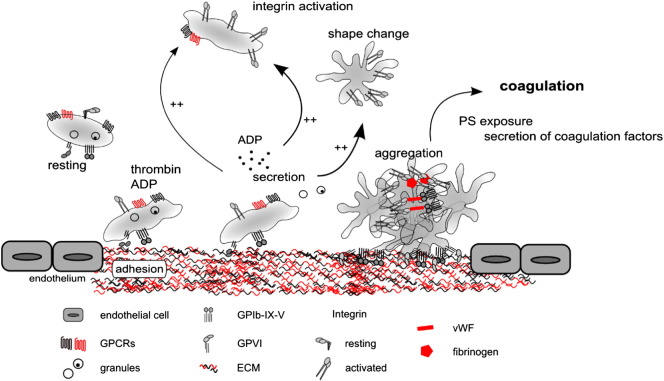
Schematic of platelet activation cascade leading to the haemostatic plug formation. Platelet adhesion to ECM components via integrin or GPVI receptors or activation with soluble agonists via GPCR receptors leads to platelet activation. One of the hallmarks of platelet activation is secretion of bioactive molecules from dense and α-granules, which can then act to activate further platelets, as well as in an autocrine manner to drive positive feedback cascade. ‘Inside-out’ signalling initiated by platelet activation also causes activation of integrin αIIbβ3. Platelets also undergo a dramatic shape change, increasing their surface area available for adhesion to ECM and to one another. Activated integrin αIIbβ3 and fibrin contribute to formation of the initial aggregate or platelet plug. Platelets also expose PS providing attachment sites for coagulation factors. The coagulation cascade contributes to the stabilisation of the thrombus.

Under physiological conditions, platelets circulate in close proximity to the vascular walls [2], but are protected from untimely activation by the healthy endothelial monolayer which provides a natural ‘barrier’ to thrombosis, as well as by the release of inhibitory mediators such as nitric oxide and PGI2 from the intact endothelium. Platelets become activated when the continuity of the endothelial layer is disrupted and the underlying subendothelial matrix is exposed, or if inflammation perturbs the endothelium. Platelet receptors then interact with collagen and von Willebrand Factor among others, which capture the platelets and induce activation signals. The initial events following platelet activation are summarised in Fig. 1.

After the initial ‘platelet plug’ is formed at the site of injury, engagement of the coagulation cascade leads to fibrin mesh formation that encapsulates and strengthens the thrombus. As well as serving as adhesion sites for coagulation factors (via surface exposure of phosphatidylserine, PS), platelets themselves are an important source of those factors (for example Factors V [3] and XIII [4,5]), and other components that regulate coagulation such as polyphosphates [6,7] and prothrombin [8].

Platelets can also become activated via G-protein coupled receptor (GPCR) signalling downstream from soluble agonists forming at the site of thrombus. Those soluble agonists, and in particular ADP released from dense granules of activated platelets, precipitate a number of positive feedback cascades leading to rapid activation of large numbers of platelets. Ultimately, an orchestrated ‘effort’ of many other secreted mediators and cells results in restoration of vessel integrity. Arguably, secretion is the most far reaching result of platelet activation, and as such can also account for functions of platelets beyond the immediate environment of the thrombus. Therefore platelet secretion is at the heart of the control of vascular integrity, in health and disease, and is the focus of this review.

## 3 Secretion in primary haemostasis

### 3.1 Dense granules

Dense granules contain a range of small molecules [9] such as ADP, ATP, GDP, 5-HT, pyrophosphate, magnesium and calcium. Historically release from dense granules was described as ‘fast’ and indeed a recent study by Jonnalagadda et al. [10] showed that the release of [^3^H]-serotonin occurred more rapidly than PF-4 from α-granules or β-hexosaminidase from lysosomes, regardless of the agonist used to stimulate platelets [10].

Small molecules released from dense granules together with synthesised thromboxane A_2_ act back on circulating platelets and contribute to the positive feedback signalling that sustains platelet aggregation. The central role of the ADP–P2Y_12_ receptor axis in haemostasis is particularly well established. Platelet adhesion on vWF, inside-out activation of integrin α_IIb_β_3_ and P-selectin expression are all defective in *P2Y_12_^−/−^* mice [11]. Recently, chemically mutagenised mice lacking Munc13-4 (*Unc13D^Jinx^* mice) that show a complete absence of platelet dense granule secretion were generated. Those animals were also found to have significantly reduced aggregation and other markers of platelet activation such as α-granule and lysosome secretion and integrin activation [12], confirming that it is the secreted ADP rather than plasma ADP that contributes to those processes. They also failed to form thrombi in the in vivo and in vitro models of thrombosis [13]. Co-stimulation with exogenous ADP could partially rescue platelet function, underlining the essential role of ADP in driving the positive feedback loop and thus the platelet primary haemostatic response [13]. Interestingly, Munc13-4 deletion did not affect intracranial bleeding following stroke induction in mice, while at the same time they were protected from stroke progression [14]. This apparent paradox hints at different roles for ADP/P2Y_12_ signalling in haemostasis and thrombosis, possibly even in different disease scenarios. The role of ADP emerges as more important and complex than initially thought and may account for some distinguishing features of thrombosis versus haemostasis. ADP/P2Y_12_ mediated signalling also enhances signalling towards procoagulant activity and thrombin activation [15].

#### 3.1.1 ADP and the ‘core and shell’ model of thrombus structure

Despite many different agonists shown to drive platelet activation in vitro to completion, it is now known that in vivo platelet activation is non-homogeneous, possibly attributable to variations in shear under physiological conditions [16,17]. α-Granule secretion and calcium mobilization have also been shown to be heterogeneous [18]. In a recent study, Stalker et al. suggested a new model of thrombus structure, taking into account that non-uniform activation pattern. They showed that two distinct populations of platelets are present in a growing thrombus in vivo: a ‘core’ of more stable P-selectin expressing platelets, and a more porous ‘shell’, containing less activated, P-selectin negative platelets [19]. While the ‘core’ seemed to be more dependent upon thrombin and local contact-dependent activation, the recruitment of platelets to the ‘shell’ was shown to be critically dependent upon ADP signalling, with P2Y_12_ inhibition significantly decreasing the size of the ‘shell’ and not affecting the ‘core’. They also show that permeability of the ‘core’ to plasma-borne molecules is limited, suggesting that efflux of platelet granule contents in that tightly packed region could be similarly limited [19,20].

The most effective anti-thrombotic treatments currently used specifically target the ADP/P2Y_12_ positive feedback cascade, blocking the P2Y_12_ receptors and thus limiting platelet activation and aggregation and the risks of pathological thrombosis, particularly in the management of coronary heart disease [21]. However, the central role of the ADP/P2Y_12_ axis in haemostasis means that abnormal bleeding often occurs in patients treated with potent P2Y_12_ inhibitors such as clopidogrel, prasugrel or ticagrelor [22]. On the other hand, the new core and shell model could account for the ability of the same agents to reduce platelet accumulation without always causing bleeding, as they would not affect the initial platelet adhesion or the size of the ‘core’ [19]. It is possible that in humans, interindividual variations in platelet activity account for those differences. Increasing our understanding of ADP/P2Y_12_ signalling may help in discovering more selective platelet inhibitors that could specifically limit thrombosis without causing bleeding.

#### 3.1.2 Polyphosphate in coagulation

Dense granule secretion is also important in coagulation. Released calcium for instance is required at several steps of the coagulation cascade and for activation of the prothrombinase pathway. Recently more insight was gained into the role of another dense granule cargo molecule, polyphosphate (polyP). PolyP is a highly anionic linear polymer that is synthesised from ATP and secreted by platelets after activation [23]. Synthetic polyP could restore defective clot formation in platelet-rich plasma from Hermansky–Pudlak patients who lack dense granules [24], suggesting a role in haemostasis. The putative mechanism involves direct polyP binding to Factor XII, thus triggering coagulation by the tissue factor-independent, contact activation pathway [24,25]. On the other hand, humans and animals lacking Factor XII were shown to have no bleeding tendencies suggesting that the contact pathway is not essential in primary haemostasis, contrary to the tissue factor pathway [26]. This would not support the idea of the polyP–Factor XII pathway being essential for haemostasis. However, more recent studies of mice deficient in hexakisphosphate 6 (IP_6_) kinase required for synthesis of polyP showed that a 3-fold reduction in polyP levels led to widespread platelet function and coagulation defects, again suggesting a role in haemostasis [6]. As well as its debatable role in Factor XII activation, polyP was also found to accelerate thrombin generation [27] and enhance fibrin clot stability [28]. Although the role of polyP and its potential in antithrombotic therapy remain largely unclear, the general consensus is that this previously under-appreciated inorganic molecule may represent an important player in the thrombotic–haemostatic environment [7]. Indeed, in 2012 two groups showed that pharmacological inhibition of polyP could prevent thrombosis without increasing surgical bleeding in mouse models [29,30], again emphasising the multifaceted role of the platelet secretome in haemostasis.

### 3.2 α-Granules

A number of α-granule cargoes, such as vWF and fibrinogen, act to propagate activation and aggregation of platelets at the site of injury. In addition, an estimated one third to a half of total α_IIb_β_3_ and one third or more GPVI receptors reside in α-granules and are trafficked to the surface of the platelet following platelet activation, further amplifying platelet aggregation [31].

#### 3.2.1 Thrombospondin and CD36 in thrombus stabilisation

In addition, a recent study identified the thrombospondin (TSP1)/CD36 axis as another pathway that may help differentiate physiological haemostasis from thrombosis. TSP1 is one of the most highly expressed proteins in platelet α-granules and unlike vWF or fibrinogen is present in plasma in very low concentrations. As shown by Kuijpers et al., knockdown of the TSP1/CD36 axis in mouse platelets leads to a defect in thrombus stabilisation without affecting primary haemostasis [32]. Initial platelet adhesion under shear in whole blood was not affected in *Tsp1^−/−^* mice, but activation as measured by PS exposure was significantly reduced. Similarly, they also showed that in the whole blood perfusion model, *Tsp1^−/−^* or *CD36^−/−^* thrombi disintegrated faster, but the thrombus size was the same between genotypes. In vivo both *CD36^−/−^* and *Tsp1^−/−^* mice showed longer time to occlusion and increased embolization in thrombosis assays. The Tsp1/CD36 axis may therefore be another contributor to the complex haemostasis/thrombosis balance.

#### 3.2.2 α-Granules in coagulation

Factors V, XI and XIII are all stored in α-granules, as is the thrombin precursor, prothrombin. Platelet derived Factor V is a potent procoagulant, thanks to higher resistance to activated Protein C-mediated inactivation than its plasma counterpart and targeted release at the site of injury [33,34]. It was also shown to explain the paradoxically mild bleeding diathesis in patients with congenital plasma Factor V deficiency, its residual secretion from platelets being able to rescue thrombin generation and thereby prevent bleeding [3]. Likewise, releasate from washed platelets, even from plasma Factor XI-deficient donors, was able to correct the clotting defects observed in Factor XI-deficient plasma in vitro [35].

Importantly, platelet α granules also contain numerous anti-coagulation cargoes. Tissue factor pathway inhibitor (TFPI), protein S, protease nexin-2 (amyloid β-A4 protein), plasmin and its inactive precursor plasminogen can all limit progression of coagulation by inhibiting or cleaving activated clotting factors or initiating fibrinolysis [31]. These apparently conflicting functions of platelets in the coagulation pathway are essential to sustain haemostatic balance and to prevent pathological thrombosis under physiological conditions.

## 4 Importance of secretion in normal haemostasis: lessons from patients

Since the primary function of platelets is haemostasis, or prevention of bleeding, it is no surprise that many patients presenting with abnormal bleeding are found to have defects in their platelet function and secretion. There are two classes of heritable bleeding disorders associated with poor platelet secretion: (i) those that result from defective platelet granule formation, leading to low numbers of platelet granules and cargo and (ii) those that arise from defects in secretory machinery, where platelets may have normal numbers of granules, but have defects in their ability to secrete them. Both of these classes of secretion deficiency have helped us to understand the role and mechanisms of platelet secretion on different levels.

### 4.1 Platelet granule formation defects

Platelets derive from megakaryocytes (MKs) — large polyploid progenitor cells that reside primarily in the bone marrow. The synthesis and packaging of granules are thought to occur mainly at the early megakaryocyte stage [36], but little is known about exact mechanisms of granule sorting and trafficking to their final destination in platelets [37]. Limited evidence suggests that, unlike most of the other secretory organelles that bud from the Golgi apparatus, both α- and dense granules may instead originate from the multivesicular bodies (MVBs)/late endosomes in megakaryocytes [38]. Most inherited platelet storage defects involve either α- or dense granules, but not both, suggesting the existence of distinct granule biogenesis pathways for the two subtypes. Understanding of congenital platelet disorders has made major contributions to our understanding of granule biogenesis as well as shed light on the roles of platelet granules in haemostasis and thrombosis.

#### 4.1.1 Dense granules

Since dense granules are lysosome-related organelles, the best described dense granule formation defects are associated with systemic lysosome-like organelle biogenesis defects [39]. Therefore patients tend to manifest with other systemic lysosome-related organelles symptoms such as innate immunity defects [40], and in both humans and related mice genetic models of disease melanosome deficiency, resulting in defective eye, skin and hair or coat pigmentation, is often present [41].

Hermansky–Pudlak Syndrome (HPS) was first described in 1976 [42], but it is only recently that advances in genomic approaches allowed for identification of the genes responsible for this disease which in turn increased our understanding of granule biogenesis in platelets. The lack of a secondary aggregation response of platelets to exogenous stimuli in HPS patients means that bleeding complications are common, although bleeding is not the most common cause of death among HPS patients. Bleeding manifestations include spontaneous bruising, epistaxis, menorrhagia, and prolonged oozing after trauma or minor surgery such as a tooth extraction [43]. Distinct subtypes of HPS are caused by defects in one of at least eight genes (HPS-1 through HPS-8) encoding the HPS proteins which interact with each other in complexes termed BLOCS 1–3 (biogenesis of lysosome-related organelles complexes 1–3) [44].

Another dense granule storage defect with much poorer prognosis than HPS is Chediak–Higashi syndrome (CHS) [45]. Again, patients lack dense granules and present with bleeding diathesis and decreased pigmentation, accompanied with severe immunologic defects and neurological dysfunction [46]. The CHS gene has been cloned and a series of mutations described, but the function of the affected protein (lysosomal trafficking regulator, LYST) remains unknown [46]. Its distinct structural domains including the BEACH (Beige and Chediak–Higashi) domain [47] suggest a function in membrane trafficking and organelle biogenesis, similar to genes affected in HPS.

Isolated dense storage pool disorders not associated with systemic defects in lysosomal-related organelles are quite common and can result in mild to severe secretion defects with or without affecting secondary aggregation, and thus presenting with varying degrees of bleeding diathesis [48]. Identification of the genes involved in those defects could potentially help understand the differences between haemostasis and thrombosis for clinical benefit (see later sections in this review).

#### 4.1.2 α-Granules

α-Granules are the storage site for many proteins including those synthesised in MK or endocytosed from plasma, with multiple functions in haemostasis and other processes described later in this article. Mechanisms of α-granule formation are less well understood. Defects of protein packaging and α-granule biogenesis in MK result in a set of heterogeneous disorders collectively known as Grey Platelet Syndrome, resulting in a usually mild to moderate bleeding disorder which can however on occasions be life-threatening [49]. In 2011, next generation DNA and RNA sequencing technologies were used by 3 groups independently to identify mutations in the *NBEAL2* (neurobeachin-like 2) gene in a large number of GPS cases [50–52]. *NBEAL2* belongs to the same gene family as *LYST* responsible for CHS, and appears to be directly implicated in α-granule biogenesis in MKs. *Nbeal2^−/−^* mice show defects in primary thrombus formation and are protected from inflammatory brain infarction following focal cerebral ischaemia [53,54]. They also have defects in tissue repair after injury, reflecting the importance of platelet α-granule secretome beyond the haemostasis/thrombosis context [53]. Another α-granule biogenesis disorder, arthrogryposis, renal dysfunction, cholestasis syndrome (ARC) is a severe multisystem childhood disorder caused by mutations in a novel Sec1/Munc18 family member VPS33B [55]. VPS33B is known to be involved in intracellular trafficking in yeast, but its role in α-granule biogenesis remains to be elucidated. Quebec syndrome on the other hand is not associated with granule biogenesis defects as such, but with degradation of α-granule cargoes due to marked increase in urokinase plasminogen activator at the megakaryopoietic stage, resulting in production of profibrinolytic platelets [56,57]. More research is needed to fully understand the genetic basis for α-granule formation and defects which could perhaps enable us to modify them for clinical benefits associated with their multifunctional cargoes.

### 4.2 Defects in secretory machinery

Relatively recently another family of congenital disorders was linked with its genetic correlates to help us understand how secretion in platelets is regulated. Familial haemophagocytic lymphohistiocytosis (FHL) patients present with an abnormal bleeding diathesis without any morphological changes to platelet granules. Unlike storage pool disorders that rarely affect both α- and dense granules, some FHL subtypes affect all granule types, suggesting a common ‘core’ mechanism to regulate secretion. In addition, systemic complications beyond platelets suggest a common mechanism of secretion in other haematopoietic cells.

**Fig. 2:**
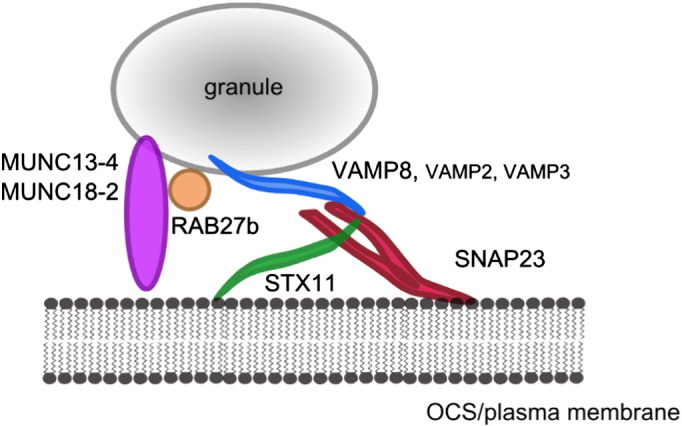
Currently accepted model of platelet secretion. Three SNARE proteins: transmembrane VAMP8, with some additional VAMP2 and VAMP3 contribution (blue), and membrane-anchored STX11 (green) and SNAP23 (red) form the core SNARE complex in platelet secretion. MUNC13-4 and MUNC18-2 (purple) are also essential for secretion, although their exact mechanism of action is not fully understood. RAB27b, along with other small GTPases (orange) is also implicated.

Indeed, platelets share their secretory machinery, centred on the *S*oluble *N*SF *A*ttachment *P*rotein *Re*ceptor (SNARE) family of proteins, with other mammalian cells. In the simplest model, the universal SNARE-mediated fusion involves transport of the vesicle to the target membrane and ‘priming’ it for release, followed by calcium-mediated conformational change in the SNARE complex that leads to the completion of membrane fusion, and eventually to the release of granule contents [58]. Four core SNARE proteins, characterised by 60 amino acid coiled-coil SNARE domains, as well as SNARE-associated proteins such as Sec1/Munc18, Munc13-4 and small GTPase families regulate that process [59], but our understanding of the machinery remains incomplete. SNAREs were first identified in platelets in 1997 [60], and since then, our understanding of the mechanisms of secretion has increased tremendously (see Fig. 2). However, some questions regarding the identity of the central ‘players’ still remain to be addressed.

In FHL subtypes 3, 4 and 5, deficiency of MUNC13-4 [61], syntaxin 11 (STX11) [62] and MUNC18-2 [63,64], respectively, leads to substantial defects in platelet granule secretion. Prior to the discovery of those genes, the mechanisms of SNAREs in human platelets were based upon treatment of streptolysin-O permeabilised platelets with functional blocking antibodies and the correlative evidence of phosphorylation of certain proteins occurring at a similar rate to secretion [65–68]. Gene knockout mouse models of a range of SNAREs (namely vesicle-associated membrane proteins, VAMPs) have also helped to identify the potentially important players [69]. However, only studies on the FHL patients provided unequivocal evidence supporting the role of those three proteins in human platelets. Even the loss of the core SNARE STX11 in FHL4 patients [62] or the knockout of VAMP8 coupled with tetanus neurotoxin (TnT-LC) treatment in mouse platelets [69], does not lead to full ablation of secretion, suggesting ranked redundancy and compensation mechanisms [69]. As discussed in more detail in our recent review (Golebiewska and Poole [59]), it is likely therefore that additional SNAREs are involved in the regulation of secretion.

## 5 Platelets — not only primary haemostasis

**Fig. 3:**
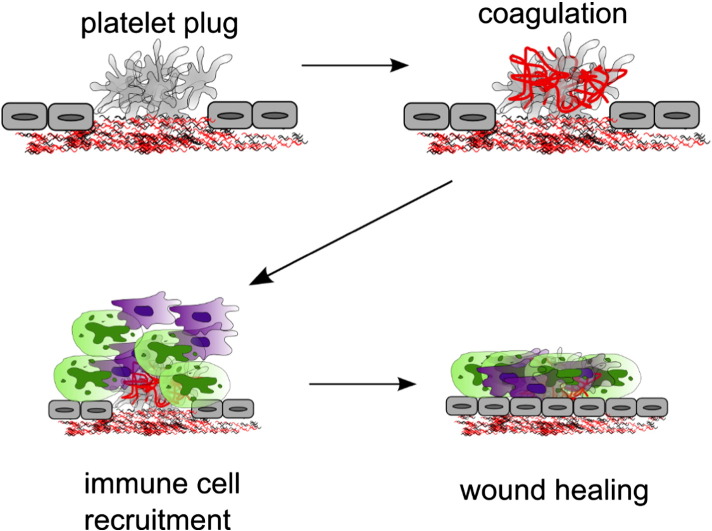
Simplified illustration of events leading to vessel injury repair. Following the initial platelet plug formation, coagulation cascade activation results in production of fibrin that reinforces the thrombus. Then leukocyte recruitment from the blood leads to an inflammatory response and antimicrobial response. Finally, wound healing and vessel wall remodelling lead to restoration of the continuity of the endothelium. Secretion of the 300 + bioactive substances from platelet intracellular stores is likely to be a major driver of these events.

The roles of platelets are not limited to initial aggregation and plug formation. The cascade of events leading to vessel repair is summarised in Fig. 3. Platelets are known to be involved at all the stages of this cascade, including coagulation, immune cell recruitment and inflammation, and wound healing, angiogenesis and remodelling. The importance of platelets in each of the steps varies in different vasculatures. In addition, our understanding of the mechanisms of thrombus formation and haemostasis comes mainly from animal models where haemostasis is initiated by physical injury to the healthy vessel. In humans, the mechanism by which ECM becomes exposed to blood flow, especially in the arterial circulation, is more often than not rupture of an atherosclerotic plaque, which also means the vessel may be inflamed, stenosed and generally unhealthy, adding to the complexity of those interactions. Therefore the mechanisms discussed here are likely to be an oversimplification of the pathological arterial thrombosis. Different mechanisms are likely to contribute to venous and microvascular versus arterial thrombosis, with alterations in sheer differentially influencing thrombus formation.

### 5.1 Platelets in inflammation

After the stable fibrin and platelet rich clot forms to stop the immediate bleeding, the further steps of the repair process begin. Close bidirectional cooperation between the haemostatic and immune systems is required to ensure restoration of normal tissue function following the injury.

In the arterial circulation, the high shear environment leads to wash out of any chemoattractant molecules from the site of the injury. Therefore platelets stably adherent to the ECM are required for ‘capturing’ circulating immune cells and recruiting them to the thrombus. Platelet α-granules mediate inflammatory responses both by expressing adhesion receptors that facilitate interactions with endothelial cells and leukocytes, and by secreting a wide range of chemokines. P-selectin translocates to the cell surface from α-granules on adherent platelets and can recruit circulatory monocytes, neutrophils and lymphocytes, inducing inflammatory responses in those cells [70]. Platelets also contain abundant chemo- and cytokines, in particular CXCL4 (PF-4) and 7 at concentrations 1000-fold greater than the plasma concentration [71]. PF-4 has been shown to drive monocyte activation and differentiation [72], as well as neutrophil adhesion [73] and monocyte recruitment to the endothelium [74].

Inflammatory cell infiltration of the arterial subendothelium, promoted by platelet cytokines, also contributes to atherosclerotic plaque formation [75]. In addition, serotonin released from dense granules upon activation by the inflamed endothelium further contributes to recruitment of immune cells to the vascular wall [76]. Transendothelial migration of neutrophils both in human and mouse models of inflammation, is at least in part mediated by a platelet α-granule-derived P-selectin and P-selectin glycoprotein ligand (PSGL)-1 interaction [77]. In addition, the mechanism by which platelets are able to guide leukocyte migration through thrombi has also recently been identified [78]. Release of NAP-2, the CXCR1/2 ligand, from platelet α-granules, leads to a chemotactic gradient inside the growing thrombus. This in turn enables leukocytes to migrate through the physical barrier which is the growing thrombus and towards the site of the injury and endothelium [78].

In addition to their essential roles in physiological responses to injury, platelet–immune cell interactions are also likely to be very important in the pathophysiology of atherosclerotic plaque formation. Modulation of platelet secretion could therefore help reduce plaque growth. For example, inhibition of platelet derived CXCR4–CCL5 heterodimer formation was shown to attenuate monocyte recruitment to inflamed vessel wall in *ApoE^−/−^* mice [79] and also reduce aneurysm formation in those animals.

### 5.2 Antimicrobial responses

Trauma to tissue or introduction of foreign objects into the vessel is the most direct way by which bacterial and viral pathogens can invade the body. Therefore it stands to reason that antimicrobial and haemostatic responses are intimately linked. Platelets are the most numerous cells first to arrive at the site of injury, and the role of platelets in combating pathogens is increasingly apparent. It has been known for many years that platelets localize and adhere to sites of bacterial lesions in the circulation, for example in endocarditis [80]. However, only in 2002 was it shown that many of the cytokines secreted by platelets have direct microbicidal properties [81], and that platelets can directly internalize pathogens [82]. At the same time, activation by pathogen-associated molecular patterns (via Toll-like receptors, TLRs) leads to the release of more cytokines, such as PF-4 or CCL5 (RANTES) which leads to recruitment of circulating inflammatory cells [83] and ensures a rapid response to infection. Interestingly, TLR-mediated platelet responses to different bacterial species vary — for example, secretory patterns of PDGF (platelet-derived growth factor) and RANTES, but not P-selectin or PF-4 differs between platelets in response to *Escherichia coli* or *Salmonella* species [84]. The increasing importance of platelets in the antimicrobial response has been reviewed exhaustively by Yeaman [85]. The precise mechanisms of the platelet antimicrobial response and differential secretion remain to be elucidated.

### 5.3 Wound healing

The final steps of the haemostatic response to external injury, or rupture of atherosclerotic plaque in the arterial circulation, lead to restoration of the integrity of the vascular wall. This process involves orchestrated proliferation and migration of smooth muscle cells (SMCs), fibroblasts and endothelial cells. Platelets' wealth of growth factors and chemokines is known to contribute to regulation of those processes. Platelet derived growth factor (PDGF) in particular is instrumental in regulating SMC proliferation and migration in both the arterial and venous circulation [86]. Neointimal hyperplasia and restenosis, such as often occurs following balloon angioplasty, were already shown to be mediated by PDGF 25 years ago [87]. Subsequently approaches targeting the PDGF signalling pathway developed but despite promise in animal models, PDGF inhibition has not yet shown to be effective in humans [88].

Another important mediator in healing and remodelling is platelet-derived SDF-1α which mediates CD34^+^ bone marrow derived progenitor cell recruitment to the injury site and their differentiation into endothelial progenitor cells [89–91]. Inhibition of SDF-1α binding to its receptor CXCR4 was shown to retard diabetic wound healing in experimental models by impairing cellular migration while concomitantly prolonging the inflammatory response [92]. At the same time, platelet-mediated differentiation of CD34^+^ progenitor cells into mature foam cells is of particular importance in atherosclerotic plaque formation [93]. SDF-1α is also a potent pro-angiogenic mediator, enhancing CXCR4-expressing endothelial cell proliferation, differentiation, sprouting and tube formation in vitro and in vivo [94–96]. Other pro- and anti-angiogenic factors such as VEGF (vascular endothelial growth factor) and endostatin are also released from platelets [97], supporting their crucial role in regulating the revascularisation of the damaged tissue.

In addition to angiogenic factors, platelets also store and secrete a number of tumour necrosis factor (TNF)-α-related apoptosis regulators such as CD95, Apo2-L and Apo3-L which can induce inflammatory responses and apoptosis in other circulating cells, as well as anti-apoptotic molecules [98]. The balance between pro-apoptotic and anti-apoptotic molecules, adequately promoting survival or eliminating the cells from the wound site, is also crucial for regulation of healing.

In summary, the increasing understanding of the role of platelet secretion in wound healing and tissue regeneration led to the development of multiple applications, especially in trauma management. Autologous platelet-rich plasma gels and growth factor concentrates are used in a number of clinical scenarios, from healing of acute skin wounds and diabetic ulcers to regeneration of tendon, ligament and nerve tissue [99]. Modulation of secretion for clinical benefit on the other hand is the area that remains to be further investigated.

## 6 Platelet secretion and disease

Platelets' wealth of potent bioactive molecules is essential for the regulation of the complex physiological processes outlined so far. However, their ability to influence other cells means that they are also central to the pathophysiology of disease. Platelet secretion can cause disease beyond the obvious thrombotic scenarios, such as atherosclerosis, or heart attacks and strokes resulting from occlusive thrombus formation in circulation. Multiple roles in conditions from cancer progression and metastasis, to chronic inflammatory conditions, sepsis and acute lung injury have also been proposed.

### 6.1 Malignancy

Early evidence for platelet function in cancer progression was originally derived from studies in a mouse model of severe thrombocytopenia [100]. In 1968, Gasic et al. reported that platelet depletion caused by neuraminidase pre-treatment led to a significant decrease in number of lung metastases following tumour inoculation [100]. It was later found, that infusion of resting, but not degranulated platelets, could rescue the tumour metastatic potential, in addition to preventing thrombocytopenia-induced tumour bleeding [101]. Thus, platelet secretion appears to be the essential permissive and protective factor for tumour metastasis.

Platelet α-granules contain a number of growth factors that are important in physiological wound healing and angiogenesis including VEGF, PDGF, epidermal growth factor (EGF), and transforming growth factor beta (TGFβ) [102]. The same molecules can be ‘hijacked’ by cancer cells to potentiate tumour survival and metastasis, which is essential for tumour survival beyond 1–2 mm in size. PDGF as a potent mitogen for mesenchymal cells including fibroblasts, smooth muscle cells, and glial cells, was a prime suspect in mediating cancer metastasis [103]. Indeed, overexpression of the PDGF receptor on tumour cells correlates with increased metastatic potential in breast cancer [104]. PDGF/PDGF-receptor interaction was also found to promote lymphangiogenesis and lymphatic extravasation in thyroid cancer [105]. Circulating VEGF, mostly derived from platelets, not only facilitates angiogenesis and increases vascular permeability, which promotes tumour cell extravasation [106], but was also recently implicated in the function of cancer stem cells and tumour initiation [107]. Platelet-derived TGFβ, in synergy with other platelet-bound factors, was recently shown to be an essential permissive factor for metastasis, specifically in epithelial–mesenchymal-like transition [108]. On the other hand, platelets also contain anti-angiogenic factors (such as PF-4, TSP-1 and endostatin) that can limit cancer survival and growth [109]. This again provides evidence for the multifaceted and complex roles of platelet secretion in pathological processes. Regulating platelet secretion could be a viable target for anti-metastatic therapies, and indeed, PF-4 is being developed as a novel anti-angiogenic cancer therapy [110].

In addition, Schumacher et al. recently showed that *Unc13d^Jinx^* mice, that lack expression of Munc13-4, were protected from lung metastases and suggested that lack of ATP secretion from platelet dense granules was responsible for that protection [111]. Although we and others [12,14,112] had previously shown a defect in α-granule secretion in these mice, consistent with an amplificatory role for secreted ADP (as described earlier in this article and Harper et al. [112]), they convincingly argue that ATP acting via P2Y_2_ receptors on endothelial cells contributes another mechanism that increases endothelial cell permeability and facilitates tumour cell extravasation, additional to the well-established pro-angiogenic functions of α-granule releasate.

### 6.2 Other conditions

Due to their ability to initiate and sustain inflammatory responses platelets are often one of the main culprits and reasons for mortality in a series of other conditions. Therefore in some cases, platelet inhibition or depletion correlates with better outcomes. The exception to that is sepsis, where a role for platelets in exuberating the disease was proposed. On the one hand, platelet accumulation in vital organs can cause inflammatory responses by platelet-mediated immune cell recruitment [113] and can lead to acute organ injury. On the other hand, disseminated intravascular coagulation leads to thrombocytopenia which in turn increases vascular permeability, normally stabilised by platelet secreted VEGF [114]. This in turn can predispose to edema, shock and organ failure that more often than not lead to death in severe sepsis [115].

The platelet P-selectin/PSGL interaction mediating platelet–neutrophil interaction in infection, is also a proposed mechanism for monocyte recruitment accompanying transplanted graft rejection [116] and blocking P-selectin was recently shown to protect mice from antibody-mediated rejection [117]. Similarly, P-selectin mediated neutrophil recruitment [118] and platelet-derived CXCL4–CCL5 heterodimer formation [119] are thought to be major pathogenic contributors to the acute lung injury.

Via their pro-inflammatory potential, platelets are also linked to inflammatory bowel disease (elevated levels of RANTES are found in Crohn's disease patients) [120], migraines (IL-1 and β-thromboglobulin are implicated in their aetiology) [121] and asthma (increased P-selectin mediated leukocyte recruitment thought to contribute to pulmonary inflammation) [122]. Therefore it is reasonable to hypothesise that many further conditions associated with systemic inflammation could be linked to platelet function or dysfunction.

## 7 So… how is it all regulated?

**Fig. 4:**
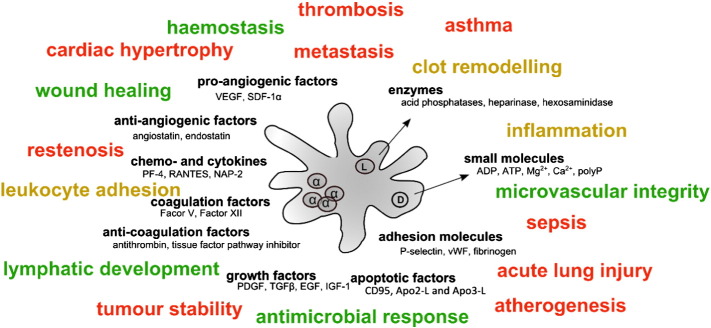
Summary of some of the platelet granule cargoes and their functional classification. α-Granules (left hand side) contain cargoes with often opposing actions (e.g. angiogenesis and coagulation-related factors), hence a mechanism(s) ensuring tight spatial and temporal regulation of secretion is likely to be in place to allow platelets to exert their many functions. Some of the physiological (green) and pathological (red) processes that are now known to be affected by platelet secretome are also listed (amber indicates both depending on the scenario). Many of those physiological and pathological processes can be affected by a combination of factors. It should be noted that although functions are assigned to each cargo here, many cargoes have multiple roles, while the roles of others still have not been fully elucidated.

Adapted from Golebiewska and Poole [59].

As outlined in this review, the roles of platelets extend beyond initial platelet adhesion and aggregation at the site of injury. Platelets are not ‘merely’ contributing to processes such as coagulation, inflammation, and antimicrobial responses or wound healing, they are arguably central and essential in the majority of those processes. As outlined, platelet secretion of 300 + molecules from intracellular stores is likely to be a major driver of those interactions (summarised in Fig. 4). Yet, clearly our current understanding of platelet secretion at the molecular level is insufficient to explain how the careful balance between all the potent mitogenic, pro-angiogenic, anti-angiogenic, pro-inflammatory, anti-inflammatory and adhesive factors released from granules under certain activation conditions is achieved. If all the contents of the intracellular stores were released in an uncoordinated manner, the thrombus growth and all the following events would be similarly uncoordinated. It seems implausible that just a handful of SNARE proteins can achieve that. Therefore several other mechanisms and avenues have been investigated recently.

One of the hypotheses put forward is that platelet granules are not uniform, and that certain factors, especially α-granule cargoes, may be differentially packaged and thus released ‘thematically’. Ma et al. observed that platelet stimulation with specific protease-activated receptor (PAR) 1 or PAR4 agonist resulted in the preferential release of VEGF or endostatin, (anti- and pro-angiogenic factors, respectively) [123]. Differential packaging of granules was a very attractive explanation for that observation, and indeed, spinning-disk confocal microscope images of resting platelets, clearly showing distinct localisation of two of the main α-granule cargoes, fibrinogen and vWF, have been published [124]. Immunogold labelling by the Italiano group also showed α-granule populations containing either endostatin or VEGF but not both [125]. More recently however, a super-resolution analysis of 15 platelet α-granule cargoes using 3D structured illumination microscopy (3D-SIM) failed to confirm any functional co-localisation [126]. van Nispen tot Pannerden et al. identified different classes of α-granules, observing snapshots of resting platelets under EM [127]. They reported a distinct population of approximately 50 nm-wide ‘tubes’ that they designated ‘tubular α-granules’. They contained fibrinogen but not vWF, as opposed to the usual, ‘spherical’ α-granules which contained both. Differential packaging of α-granule cargo remains an attractive explanation for the observed separation of pro- and anti-angiogenic releasates, but the difficulties associated with platelet research mean that we still lack definitive confirmation of this hypothesis. No similar studies on dense granule secretion exist.

In 2012, the Whiteheart group performed a systematic quantification of granule secretion using micro-ELISA arrays for 28 distinct α-granule cargo molecules in response to 4 different agonists [10], quantifying the kinetics of secretion of each cargo. This was the first time that kinetics of secretion were measured — no conclusive evidence for the ‘fast’ dense granule, ‘slower’ α-granule and ‘slowest’ lysosome secretion model previously existed. They found that not only secretion of different cargoes varied depending on the agonist used, but that there was also an overlap in secretion kinetics of cargoes of opposing functions. In conclusion, they proposed that the kinetics of release could not independently explain differential secretion and granule shape, proximity to the membrane or cargo solubility is likely to influence the kinetics [10]. It is also likely that different SNARE complexes could drive the fusion with different kinetics — another reason to question the ‘one size fits all’ SNARE complex in platelets that is currently accepted (SNAP23–VAMP8–STX11). SNARE redundancy and functional specialisation have been described in other cells. For example two endosomal SNARE complexes exist, with one mediating the early to late endosome transport, while the other — late endosome to surface step [128]. Similarly, human neutrophils have several different SNAREs that regulate secretion of specific granules [129]. Therefore it is possible that differential secretion of cargoes in platelets is also mediated by not one, but several different SNARE complexes. In addition, it is thought that platelet granules may fuse with each other or with the open canalicular system (OCS) rather than directly with the plasma membrane, which could accounts for different secretion rates depending upon the stimulation conditions [130]. Signalling cascades between agonist stimulation and SNARE-mediated secretion events also remain largely unknown. The accepted model of platelet signalling principally converges on the increase in cytosolic calcium to drive the cellular machinery upon activation, but the signalling downstream of receptors is likely to diverge at some point to appropriately drive different secretory events. Again, the nature of platelets means that studying cell signalling in real time is problematic. Finally, of course, dissecting platelet function in vitro or in animal models of thrombosis does not necessarily reflect their function in human health and disease, and this remains a major challenge for future research. In summary, our understanding of platelet secretion is increasing, but remains rudimentary at the moment with no clear answers to the question of how the remarkable complexity is achieved.

## 8 Conclusions

Platelet secretion is undoubtedly of pivotal importance for regulating not only canonical platelet functions, but also mediating their impact on other cells, and understanding platelet secretion may have consequences reaching far beyond the ‘platelet field’. Yet platelets remain less well studied in terms of mechanisms governing their function, relative to other secretory cells such as neuroendocrine or immune cells. In this review we have shown examples of the ways in which platelet secretion is essential for sustaining haemostasis and ensuring an adequate response to injury, but also how the same mechanisms may mediate pathological responses. This means that any approaches targeting platelets or platelet secretion have to be carefully tailored for each specific application. Dissecting mechanisms of differential secretion of different cargoes may be helpful in designing therapies that can avoid currently common bleeding side effects while limiting pathological inflammation, tissue hypertrophy or cancer cell metastasis.

## Conflict of interest statement

None.
